# “Research Jam”: Engaging patients and other stakeholders through human-centered design to improve translational research

**DOI:** 10.1017/cts.2022.498

**Published:** 2022-11-14

**Authors:** Sarah E. Wiehe, Courtney M. Moore, Dustin O. Lynch, Gina Claxton, Nerissa S. Bauer, Helen Sanematsu

**Affiliations:** 1 Children’s Health Services Research, Department of Pediatrics, Indiana University School of Medicine, Indianapolis, IN, USA; 2 Community Health Partnerships, Indiana Clinical and Translational Sciences Institute, Indiana University, Indianapolis, IN, USA; 3 NSB Consulting LLC, Carmel, IN, USA; 4 Herron School of Art and Design, Indiana University Purdue University Indianapolis, Indianapolis, IN, USA

**Keywords:** Patient and stakeholder engagement, human-centered design, research barriers, translational research, participatory design

## Abstract

Effective stakeholder engagement increases research relevance and utility. Though published principles of community-based participatory research and patient-centered outcomes research offer guidance, few resources offer effective techniques to engage stakeholders and translate their engagement into improvements in research process and outcomes. The Indiana Clinical and Translational Sciences Institute (Indiana CTSI) is home to Research Jam (RJ), an interdisciplinary team of researchers, project management professionals, and design experts, that employs human-centered design (HCD) to engage stakeholders in the research process. Establishing HCD services at the Indiana CTSI has allowed for accessible and innovative stakeholder-engaged research. RJ offers services for stakeholder-informed study design, measurement, implementation, and dissemination. RJ’s services are in demand to address research barriers pertaining to a diverse array of health topics and stakeholder groups. As a result, the RJ team has grown significantly with both institutional and extramural support. Researchers involved in RJ projects report that working with RJ helped them learn how to better engage with stakeholders in research and changed the way they approach working with stakeholders. RJ can serve as a potential model for effectively engaging stakeholders through HCD to improve translational research.

## Introduction

Clinical and translational research increasingly embraces stakeholder perspectives in research design, implementation, and results dissemination [1]. The translational research agenda advocates for a team-based approach that includes stakeholders [2]. The Patient-Centered Outcomes Research Institute (PCORI) funds research to improve the quality and relevance of available evidence to help patients and other stakeholders make more informed health decisions (https://www.pcori.org/about-us/our-story).

Though consensus is growing about the benefits of greater stakeholder participation in translational health research, the translational science of stakeholder engagement is more nascent. We posit that human-centered design (HCD) is a promising approach. In this paper, we describe how the Indiana Clinical and Translational Sciences Institute’s (Indiana CTSI) HCD service, Research Jam (RJ), improves health research’s relevance, effectiveness, and sustainability through innovative approaches to stakeholder engagement. We aim to guide others interested in pursuing similar work.

## Stakeholder Engagement in Health and Clinical Research

The premise is that translational research can better address the needs of patients and stakeholders by involving them in the research process [3]. At least three systematic literature reviews have examined the potential impact [4–6]. Findings indicate increased study enrollment and retention [4–6], more relevant research methods, questions, materials and outcomes [4,6], and improved dissemination through more meaningful content and greater access to appropriate social networks [4]. Stakeholders and researchers both worry, however, about the risk of “tokenistic” engagement in which stakeholders are involved only enough to fulfil an engagement requirement [7].

To guide meaningful stakeholder participation, PCORI established standards for patient-centered outcomes research: engage people representing the population of interest and other relevant stakeholders appropriately; identify, recruit, and retain study participants who represent the spectrum of the population of interest; use patient-reported outcomes when patients or people at risk of a condition are the best information source for outcomes of interest; and support the dissemination and implementation of study results [8]. PCORI also developed a “rubric” for operationalizing stakeholder engagement over the research trajectory [3,9]. For instance, in study planning, patients and other stakeholders may develop research questions and outcomes of importance; in study implementation, they may develop or revise study materials and protocols and participate in recruitment or data collection; and in disseminating results, they may develop a dissemination plan and identify appropriate partners [3,9].

Despite perceived benefits of engagement and big-picture guidance from institutions like PCORI, specific techniques are needed to effectively gather and translate non-researcher stakeholder input.

## Research Jam’s Beginnings

### Building the Team

The Indiana CTSI’s HCD team was founded by a health researcher (SEW) and a designer (HS) after seeing the fit between HCD and CBPR within their own research collaboration [10]. RJ’s approach was initially implemented as a patient engagement core within an Agency for Healthcare Research and Quality (AHRQ) grant (R24HS22434, PI: Carroll) and applied as a longitudinal stakeholder engagement service for four separate studies supported by this center grant [11]. The core successfully tailored these four studies to be more relevant to stakeholders, including engaging stakeholders to revise the study design, develop novel measures, address recruitment and retention challenges, and disseminate findings to the stakeholder audience, while retaining scientific rigor. This success generated demand from investigators beyond the AHRQ grant, propelling RJ to become a service core supported by the Indiana CTSI.

Today, the RJ team includes eight members: two scientific directors (including SEW, a pediatrician and public health/health service researcher), an associate director of operations (GC), four designers with training in visual communication and HCD (including CM, DL), and a project coordinator.

### Human-centered Design

The design landscape is broad, encompassing several disciplines with overlapping practices and values. The core values of the broader design field in which RJ lives are empathizing with users, working collaboratively, visualizing/prototyping to externalize ideas and gain insights, tolerating ambiguity and failure, respecting intuition, and strategically employing divergent thinking (considering multiple alternatives) and convergent thinking (recognizing patterns and relationships) [12,13]. Within this broad framework, two disciplines underpin RJ’s work: HCD and participatory design (PD).

*Human-centered design* originated in ergonomics, computer science, and artificial intelligence and focused on interactions between pre-defined products/services and their users [14]. HCD evolved to include users early in the process and to develop products/services from insights gained during interactions with users (rather than just ergonomic or human factors best-practices). Today HCD is based “on [using] techniques which communicate, interact, empathize, and stimulate the people involved, obtaining an understanding of their needs, desires, and experiences which often transcends [what] people…actually realized” [14]. Designers and other internal stakeholders drive the design but are informed by the knowledge gathered about end users. Increasingly, HCD is addressing challenges in health and healthcare [15–18].

*Participatory design* emerged to create more usable and acceptable computer systems and to advocate for more user participation in the decisions affecting computer system design and use [19], though modern PD has expanded beyond computer system design [20]. PD positions end users as experts and decision-makers because they alone experience using the product or system to accomplish tasks in the ultimate context of use [19]. PD recognizes that meaningful stakeholder participation requires considered methods and has developed a robust selection that can be utilized and adapted [20].

**Fig. 1.**
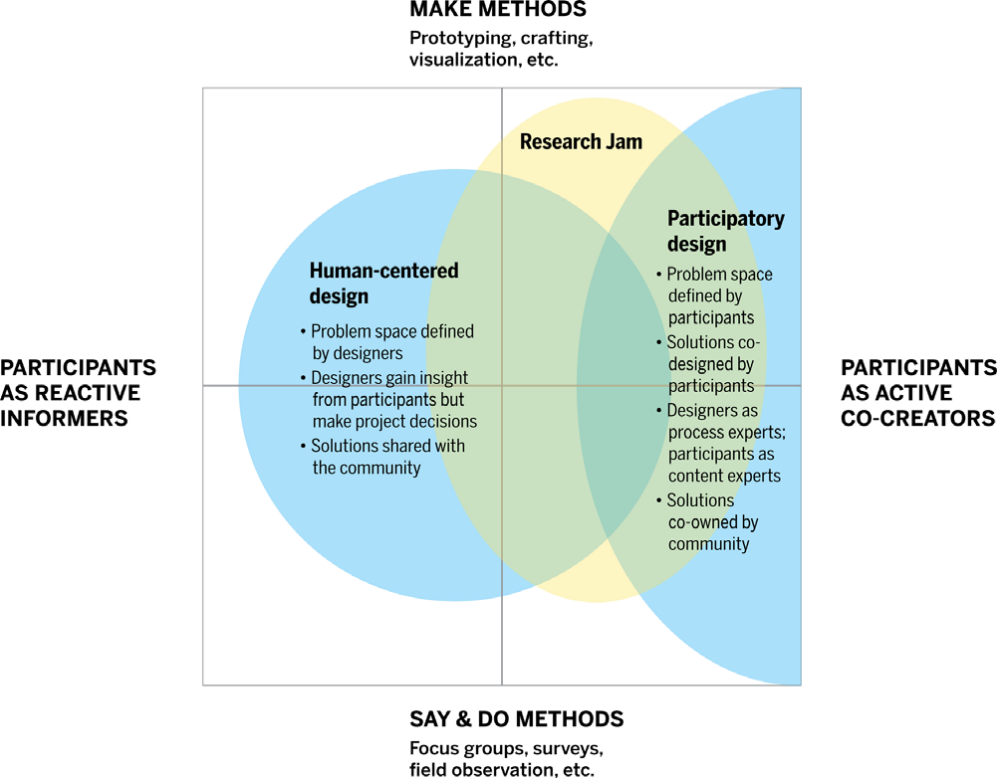
Map of Research Jam in the context of human-centered design and participatory design.

In theory, HCD and PD have many overlaps. In practice, however, HCD typically includes users less comprehensively or powerfully than PD does (see the *x* axis in Fig. 1 – adapted from a map created by PD researcher Elizabeth Sanders [21]). The key differences are whether or not the user is involved in defining the problem space (the issue to be investigated and solved) and in generating solutions. In HCD, often designers are inspired by the needs of users and are informed by human factors, but define the problem space and envision solutions without direct stakeholder participation. When a participatory approach is employed in HCD, it is typically later in the process (e.g., a workshop allowing stakeholders to envision solutions). Stakeholders function as sources of information and inspiration for designers as needed throughout the process, and designers function as design process experts and decision-makers. In PD, the designers facilitate stakeholder participation throughout the process by creating an environment where users feel empowered to propose ideas [19]. As part of this process, stakeholders function as experts in relevant experiences as well as fellow decision-makers. Designers function as experts in facilitating the design process as well as form-makers who help make the team’s ideas concrete. A similar spectrum of participation is present in implementation of community-engaged research (CEnR) in that stakeholders are not always engaged in every stage of research, specifically the front-end objective-setting phase of the research [22,23].

RJ is in a unique position. Ideally, RJ would involve stakeholders in a PD process that empowers them to co-design from start to finish. In a health research context, however, this process is difficult for several reasons [15]. Researchers typically define the problem space and/or envision the solution, which places stakeholders in a more HCD (sources of information and inspiration) rather than PD (co-designers) process. In working with clients, RJ advocates for the benefits of including stakeholders in a more participatory manner across the research process, but often problem spaces and design solutions must be defined before funding is sought and secured. CBPR faces the same tension “of selling a process without completely specifying all the outcomes beforehand, often troubling for researchers, health professionals, and community members, as well as funders” [24]. A key strength of design is its ambiguity tolerance. Though funders and researchers may struggle with undefined outcomes, designers generally embrace them and utilize methods to engage stakeholders earlier in the process when the research outcomes are undefined.

Ultimately, RJ employs an overall HCD-style approach with as much PD as allowable by the specific research situation (based on investigator preferences and funding context). In many cases, individual researchers’ first RJ projects have defined outcomes and more limited stakeholder involvement and subsequent projects are more open ended and allow for a more PD approach.

## Research Jam’s Process

RJ can assist throughout the research process—planning the study, conducting the study, and disseminating results. RJ works in collaboration with the client, who is typically a principal investigator (PI). The process includes four phases.

### PHASE 1: Develop a Research Plan

The PI approaches a RJ director to assess the fit of the project objectives with RJ’s services. At this “handshake meeting,” the director and PI discuss the scope of work, timeline, and budget. The director introduces principles of HCD and explores how this approach to stakeholder engagement could meet the project objectives. If the PI opts to proceed with RJ services, s/he completes a survey, sharing background information on his/her research and his/her current understanding and valuation of CEnR, stakeholder engagement and HCD. At a subsequent “discovery meeting,” a RJ scientific director, RJ director of operations, and at least one RJ human-centered designer (HCDer) meets with the PI to (1) frame the study objective(s) and (2) determine stakeholder populations. RJ HCDers then meet to determine recommendations for (3) the number and type of “jams” (interactions in which stakeholders engage in HCD/PD activities) needed to meet the objective(s), and (4) the best activities (see below) to use in each jam. RJ drafts a project agreement that the PI and RJ director sign. The RJ project coordinator then handles all regulatory components of RJ’s involvement, including consent development, participant incentive plans, participant privacy and protection considerations, and Institutional Review Board (IRB) approval. The study PI is generally responsible for recruiting stakeholders for jams, though RJ often consults on recruitment approaches, designs communication strategies, and refers to other Indiana CTSI recruitment resources.

Engagement activities for each jam are customized from existing HCD tools or newly developed as needed [25,26]. The choice of tools is based first on the specific objective(s) and, second, on the type of knowledge to be discovered: explicit, observable, tacit, or latent. *Explicit* knowledge (that which people can easily access, express, and share) and *observable* knowledge (that which can be learned by watching something being done) are commonly gathered in research using methods that gather what people “say” and “do” such as focus groups, surveys, or field observation. HCD gathers these through similar methods. HCD also includes methods for gathering *tacit* and *latent* knowledge. *Tacit* knowledge is what people understand intuitively but cannot easily access and express in words—for example, the feelings someone has about a subject [27]. *Latent* knowledge is knowledge not previously known by individuals or groups that, when discovered, creates new understanding. Latent knowledge is rarely discovered in isolation, but rather is typically uncovered through interactions with others—for example, as with an innovation or in discovering a new process [28]. Because tacit and latent knowledge cannot be easily expressed in words, eliciting them requires facilitating the expression of ideas through other forms of externalization such as making, something at which designers are particularly adept [27]. The *y* axis in Fig. 1 shows the say and do versus make methods and how these relate to HCD/PD disciplines and RJ’s work.

RJ chooses tools from one or more of three categories (i.e., explore, create, and test) based on the research objective and subobjectives. RJ HCDers assemble tools into an agenda for an in-person or virtually facilitated jam or a set of self-directed activities, depending on the tools and on the feasibility of assembling stakeholders. Given constraints relating to the COVID-19 pandemic, virtual jams have been deployed more regularly and have proven effective in engaging stakeholders and generating rich insights. Further, this has allowed for broader engagement geographically which has been advantageous for rare conditions and hard-to-reach populations.

*Explore* tools (eliciting explicit, observable, tacit, and latent knowledge) uncover stakeholder experiences to, for example, elucidate patient-centered outcome domains or understand strengths and weaknesses of a service experience. Techniques include mapping, gaming, and collage. For example, RJ uses experience mapping in which participants draw out their movements and interactions during a specific experience [29]. RJ applied a novel twist to experience mapping using turn-based board game mechanics (*Chutes and Ladders*). This “gamified” version produced meaningful insights into the hospital experience post-overdose [30].

*Create* tools (eliciting tacit or latent knowledge) assist participants in externalizing and refining design ideas, generating strategies for solving defined challenges, or creating low-fidelity prototypes (i.e., rough, early-stage versions) for solutions like intervention components or study tools. A final design is often developed iteratively over several jams. Techniques include prototyping and storyboarding [31]. For example, RJ regularly uses “design charrette,” an iterative, collaborative technique in which individuals or small groups create prototypes that are refined by new stakeholder group(s) [26].

*Test* tools (eliciting explicit or observable knowledge) assess how well design solutions work and identify opportunities for improvement. Tools include experience simulation [25] and usability testing [26]. Testing often occurs at various stages of refinement until a final product is designed (e.g., video script, recruitment tool, educational booklet, or service prototype).

### PHASE 2: Execute the Research Plan

Stakeholders attend a jam at a convenient location or using digital tools and receive a gift card, meal, and parking voucher as needed. Stakeholders are consented either by the research coordinator or via online consent. Two or more RJ HCDers facilitate the jam or monitor participation in self-directed tools. A representative from the client’s team may observe if there is no reason to believe his/her presence will discourage participants’ openness.

RJ asks stakeholders to evaluate each jam through a short survey and invites them to join RJ’s e-newsletter to hear about study results and opportunities to work together again.

### PHASE 3: Analyze and Create Deliverables

RJ meets with the PI post-jam to share key takeaways, hear the PI’s reactions, and discuss next steps. Following qualitative analysis, RJ delivers a final report outlining data collection and analysis methods, findings, and recommendations. RJ may then begin the development of the next jam or create final designs based on the recommendations.

### PHASE 4: Implement and Share

During a final meeting, RJ and the PI develop an implementation and dissemination plan (e.g., blog posts, publications, and community events). At project completion, RJ provides the PI a closeout survey to evaluate satisfaction with services and any change in the PI’s understanding and valuation of using CEnR and HCD approaches.

## Research Jam Case Studies

### Attention Deficit Hyperactivity Disorder (ADHD)

The TEACH (Tailoring Education for ADHD and Children’s Health) Program is a primary care-based family intervention to improve ADHD-related parent–child interactions and family management skills. The PI (NB) proposed to revise TEACH tools to better facilitate family achievement of program goals. This work supported a randomized controlled trial investigating the acceptability of ADHD group visits [32].

One TEACH goal is to facilitate parent tracking of ADHD treatment progress and increase positive parent–child communication around ADHD treatment. RJ hosted an explore jam, in which experience mapping was used with parents of children with ADHD to understand routine ADHD parent–child interactions and discovered the following:

1. Parents were proud when they successfully used positive parenting techniques to manage bedtime challenges (e.g., play money for positive behaviors and tickets for negative behaviors).
2. School attendance and busy family schedules meant parents were limited to interactions before and after school.
3. Parents liked involving their children in day-to-day activities that taught their children cooperation. For example, when experience mapping grocery shopping, parents described involving their children in finding items at the store or in putting groceries away at home.

After synthesizing these findings, RJ revised the existing TEACH ADHD treatment chart. The original chart focused on medications, behavior therapy, and school supports, but gave medications the most visual weight. The revised chart added positive parenting techniques and gave all four treatment categories equal visual weight to reinforce to families that all treatment strategies matter and to recognize positive parenting efforts. The revised chart also successfully condensed a great deal of information onto one page.

**Fig. 2.**
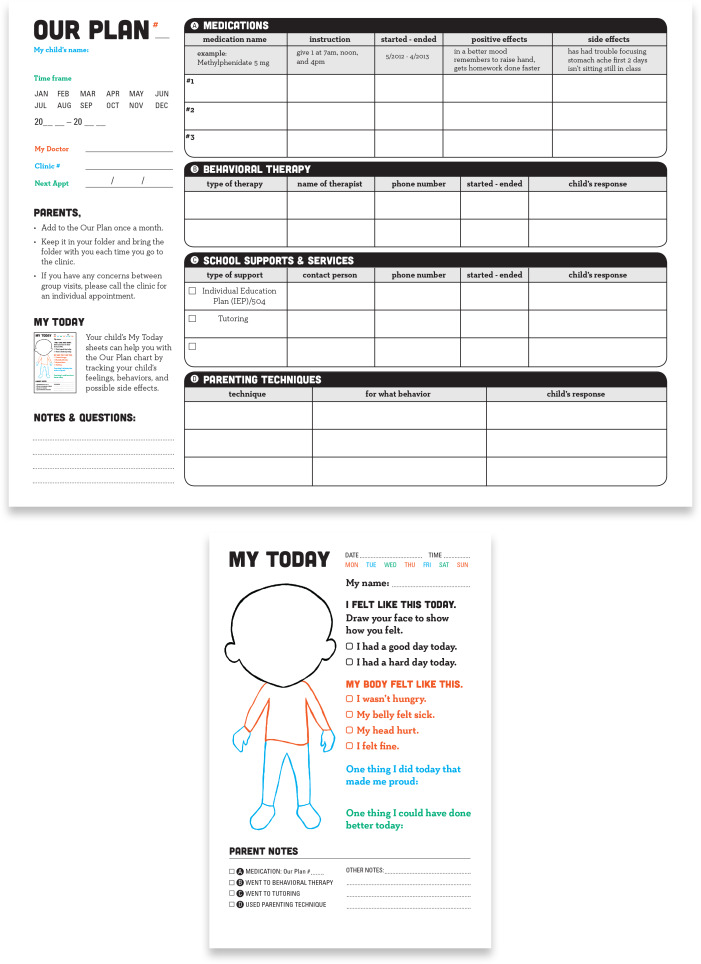
ADHD child’’s symptom tracker and treatment chart.

Moreover, RJ designed a daily mood and symptom tracker notepad for the children to (1) increase symptom-reporting accuracy, given parents spent most of the day apart from their children and (2) empower children to participate in their own care and treatment. RJ designed a magnetic (for easy refrigerator display) pad of 31 tear-off sheets that allowed the child to reflect daily on his/her general mood and any medication side effects (new knowledge children gain during TEACH sessions). The parent could easily look across several completed sheets and make notes in the corresponding color-coded sections on the treatment chart, which they could share with the child’s doctor (Fig. 2).

### Type 1 Diabetes Mellitus (T1DM)

Pediatric T1DM is a chronic condition requiring patients and parents to co-manage treatment and monitor dietary habits and blood sugars. The PI aimed to transform a traditional behavioral contract—typically used by patients and endocrinologists to outline treatment goals—into one used by teens and parents to establish co-management strategies [33]; the PI tested the resulting clinical intervention in a study of teens with T1DM [34].

RJ hosted an explore jam to facilitate discussion between teens with T1DM and their parents to understand how T1DM affects quality of life, what aspects of teen/parent conflict the contract could cover, and what negotiation tactics the contract might employ. First, participants played a game in which teens and parents anonymously responded to the question “How does diabetes most impact your life?” The facilitator read each answer aloud and participants guessed whether a teen or parent wrote it. RJ designed the empathy-raising activity to identify key challenges unique to teens or parents and key challenges shared by both groups. Then teens and parents moved to separate rooms, and each group discussed which parts of T1DM management caused conflict with the other group. Facilitators captured answers on flipcharts that they swapped between rooms so that teens could discuss how they would address the conflicts and behaviors the parents brought up and vice versa. This process uncovered additional content for the contract, areas of potential conflict due to opposing viewpoints, and negotiating strategies teens and parents use.

**Fig. 3.**
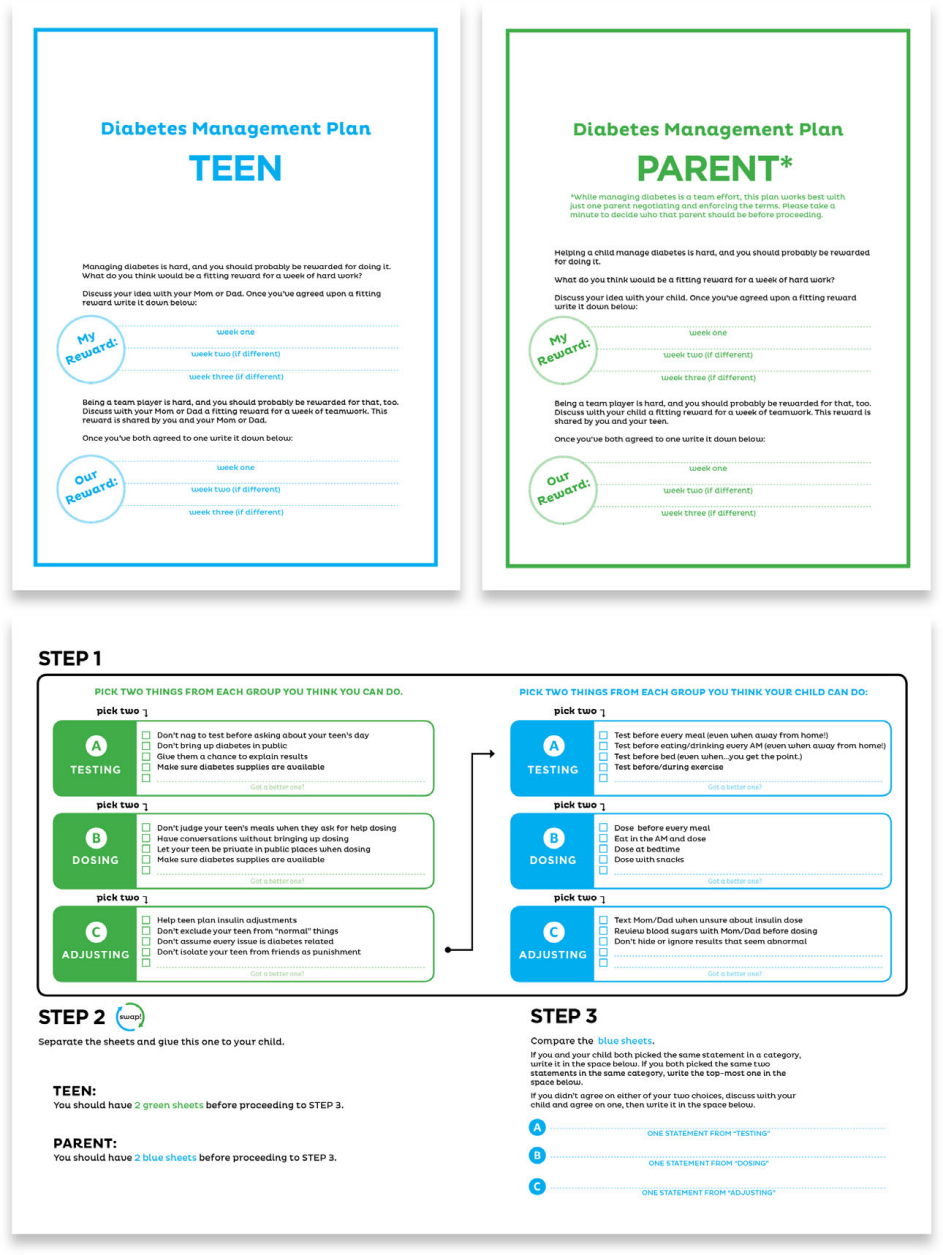
Living contracts for adolescents with type 1 diabetes [blue] and their parents [green].

Next, RJ created a prototype and then hosted a test jam to observe the prototype in use and ask follow-up questions. Because teens and parents would use the contract together, they evaluated the prototype together. Findings were integrated into a “living” contract which allowed teens and parents to express the main concerns they wanted addressed, to facilitate negotiation, to incentivize cooperation, and to visualize the terms [33]. The tool’s purpose was to help teens and parents relate more as collaborators than as adversaries (Fig. 3).

RJ has completed projects addressing various research challenges including identifying patient-centered outcomes [35,36], developing interventions [37,38], improving research study recruitment [39] and experience [40] to improve research outcomes, and communicating health information to the public [41].

## Four Key Takeaways

First, RJ’s experiences reflect the demand within the Indiana CTSI for a “non-tokenistic” connection with patients and community members. RJ has expanded (to date) from four to seven team members and manages a portfolio of multiple funded projects at any one time. The service core is attractive to investigators who receive refined “turn-key” design deliverables and who find that HCD enhances their grant applications.

Second, researcher-designer collaboration is doable, but there are challenges to overcome. RJ emerged from collaboration between authors at Indiana University School of Medicine (SEW) and the Herron School of Art and Design (HS). HCD faculty at a local art or business school can provide HCD expertise as well as connections to local talent, including both practicing HCDers and HCD students. Beginning with a small project can help partners better understand each other’s practices and mindsets, decide if further partnership is desired, and adjust processes for better cohesion. HCDers may find some of the processes in academic research foreign, such as more prescribed project outcomes than is typical in HCD and the more limited flexibility that comes with working with an institutional review board. Similarly, academic researchers may find the ambiguity HCDers allow—even require—as part of the process to be frustrating and may find some HCD methods to be unorthodox and difficult to describe in academic papers and grant proposals. RJ has piloted a HCD “bootcamp” for RJ clients to help them better understand the HCD process. RJ also writes detailed reports of its methods and findings for each project, and RJ team members are actively involved as authors on academic papers written by clients given their integral role in both the design and implementation of the work and to help with describing the HCD components. A key tenet to the approach is to not be afraid to make mistakes, especially in the early stages, as failing early (and often) offers the opportunity to iterate and often generate viable solutions as compared to an approach that is more cautious. From an institutional standpoint, because this type of work is not well represented in academic settings, RJ has had to work to build a structure within which this work is supported. We have built relationships with institutional leadership and finance to obtain support and buy-in, with an agreement to increase outside grant-funded support over time. Though RJ was internally supported in full when initially launched as an Indiana CTSI service, it is now primarily funded by extramural grants (>80%). In addition, RJ has worked with human resources to better explain the work we do so that RJ HCD team members are appropriately classified in the job system at our institution (as researchers rather than graphic designers). Finally, RJ has worked with our institutional review board to improve their understanding of and comfort with our methods, which were new to them. Working on a project to help design our institutional review board’s concise consent template (a short summary at the top of long consent forms) was a partnered effort that relayed our approach and built trusted relationships.

Third, this collaboration helps fulfill the CTSAs’ mission to improve the health of patients and communities. HCD translates between the medical community and their “target communities” to identify shared relevant goals, in turn affording better patient and provider decision-making. By working with study investigators and stakeholders through HCD, RJ is able to elicit salient stakeholder-centered input and translate it into tangible insights and deliverables. In each of the two case studies, RJ increased the relevance and effectiveness of study recruitment, materials, and outcome measures. As a busy service core, RJ has not yet formally assessed the core’s effectiveness, but has made progress in describing processes and outcomes within specific projects [42–44] and has heard anecdotal accounts of the work’s impact. For example, as part of a randomized controlled trial of surgical approaches for ureteropelvic junction obstruction thought impossible to recruit for, RJ assisted in the co-design with parents of a study information brochure and video, resulting in a recruitment rate of 92% (11 of 12 families approached over 16 months) [45]. RJ collects client and participant satisfaction measures to better understand effects on clients, stakeholders, research processes, and tools, and—more importantly, but more challenging—health outcomes. Past and present clients reported in a recent survey and interviews conducted by a non-RJ-affiliated student researcher that working with RJ changed the way they approach working with stakeholders and helped them learn how to better engage with stakeholders. One client expressed: “A lot of times, we tailor the patient to the needs of the study. And Research Jam really turns that upside down because we’re not tailoring anything to the needs of the study, we’re tailoring to the needs of the participants.”

**Fig. 4.**
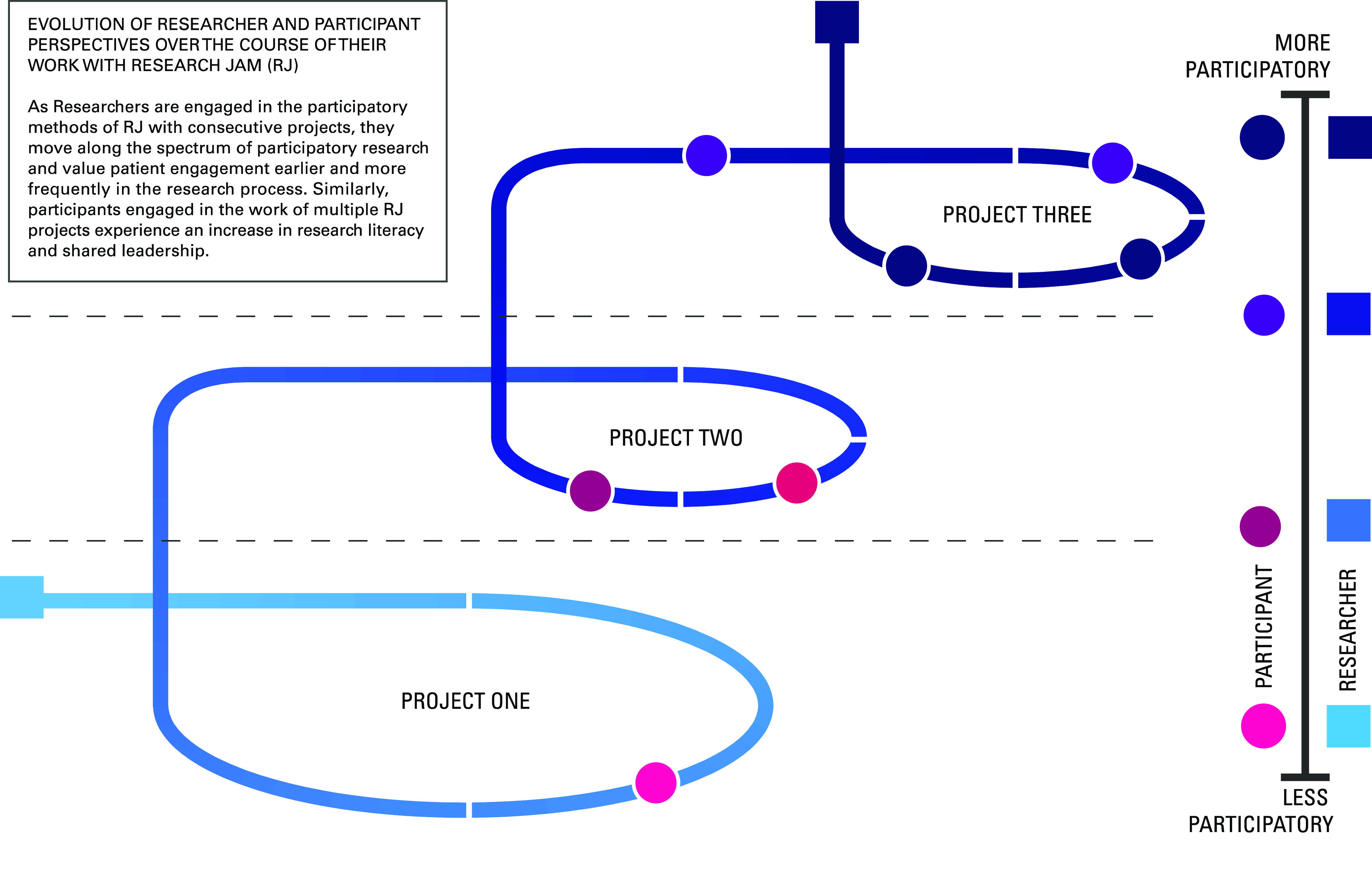
Evolution of researcher and participant perspectives over the course of their work with Research Jam.

Lastly, RJ’s work changes people. Stakeholders share stories and viewpoints that are valued by researchers and clinicians and, as a result, become more empowered. In some cases, they develop and implement their own research questions, an important first step to true CBPR and PD. Researchers, especially those with longer term or repeat RJ projects, experience the tremendous value of patients’ insights and experiences in shaping their research. Treating both researchers and stakeholders as experts helps balance the power differential that often exists. Many RJ client researchers have shifted more of their research to be community- and stakeholder-engaged and seek stakeholder involvement earlier in their research endeavors [33]. Over time, stakeholders and researchers approach a true CBPR/PD partnership (Fig. 4).

## Conclusions

This innovative HCD approach to partnering with stakeholders to improve health and clinical research is feasible, sustainable, and desirable, as evidenced by the continued funding success of RJ-involved grants, repeat clients, and the rapid growth of the RJ team and project portfolio. It is anecdotally effective at improving research relevance to stakeholders, increasing researcher valuation of community engagement, and increasing the effectiveness of research processes. HCD can be a powerful methodology in community-engaged and translational research, and RJ hopes to inspire other teams to consider this approach.
